# Trans-generational Effects of Early Life Stress: The Role of Maternal Behavior

**DOI:** 10.1038/srep04873

**Published:** 2014-05-02

**Authors:** Claudia Schmauss, Zoe Lee-McDermott, Liorimar Ramos Medina

**Affiliations:** 1 Department of Psychiatry and Molecular Therapeutics, Columbia University; 2 New York State Psychiatric Institute, New York, NY 10032

## Abstract

Using a rodent paradigm of early life stress, infant maternal separation (IMS), we examined whether IMS-triggered behavioral and epigenetic phenotypes of the stress-susceptible mouse strain Balb/c are propagated across generations. These phenotypes include impaired emotional behavior and deficits in executive cognitive functions in adulthood, and they are associated with increased acetylation of histone H4K12 protein (acH4K12) in the forebrain neocortex. These behavioral and epigenetic phenotypes are transmitted to the first progeny of IMS Balb/c mothers, but not fathers, and cross-fostering experiments revealed that this transmission is triggered by maternal behavior and modulated by the genetic background of the pups. In the continued absence of the original stressor, this transmission fades in later progenies. An adolescent treatment that lowers the levels of acH4K12 in IMS Balb/c mice augments their emotional abnormality but abolishes their cognitive deficits. Conversely, a treatment that further elevates the levels of acH4K12 improved the emotional phenotype but had no effects on the cognitive deficits. Moreover, treatments that prevent the emergence of either emotional or cognitive deficits in the mother also prevent the establishment of such deficits in her offspring, indicating that trans-generational effects of early life stress can be prevented.

Early life stress can trigger changes in gene expression and behavior that persist into adulthood. It is widely recognized that early life stress is a risk factor for several psychiatric illnesses and one of the strongest predictors of negative mental health outcomes, including poor treatment responses and suicide^1^,^2^,^3^. About 1.5% of the population of the United States between the ages of 0 to 17 years have a documented history of early life stress, either in form of childhood neglect (~60%), physical abuse (~15%), or sexual abuse (~10%)^4^. However, the impact of an individuals' early life stress experience on society is potentially larger if the persistent abnormalities triggered by early life stress are propagated across generations. At present, however, little is known about trans-generational effects of early life stress, especially about maternal and paternal effects on subsequent generations.

Animal models afford major advantages for the study of early life stress-triggered changes in gene expression and behavior and their potential propagation across generations. Studies on the inbred mouse strains Balb/c, for example, showed that a powerful rodent paradigm of early life stress, infant maternal separation (IMS; a daily 3 hour separation of mothers from their 2 to 15 day-old pups), triggers persistent changes in emotional and cognitive behavior as well as changes in forebrain neocortical gene expression that are not found in the genetically different inbred strain C57Bl/6^5^,^6^,^7^,^8^. In Balb/c mice, one prominent outcome of the changes in gene expression is reduced activity of several members of class I and II histone deacetylases (HDACs). This leads to increased acetylation of histone H4 protein, especially increased acetylation of histone H4K12 (acH4K12), an epigenetic mark of open chromatin and facilitated gene transcription^8^. The discovery of this epigenetic mark raised the possibility that it is functionally linked to some of the most prevalent adult behavioral phenotypes elicited by early life stress and perhaps also transmitted across generations. To test this, we examined the behavioral and molecular phenotypes of progenies of Balb/c mothers and fathers exposed to early life stress. We found evidence for a germline-independent transmission of behavioral and epigenetic marks of early life stress that is mediated by maternal behavior during postnatal care of her pups, modulated by the genetic background of pups, and susceptible to intervention during adolescent development of future mothers.

## Results

### The emotional phenotypes of the progenies of IMS mothers and fathers

**Figure 1:**
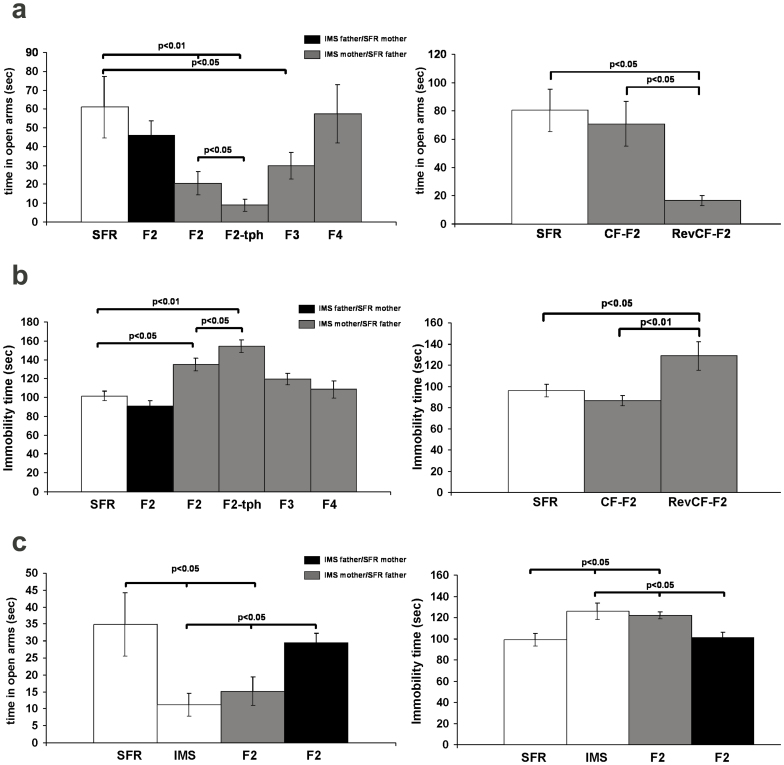
Maternal transmission of the IMS-triggered emotional phenotype. (a) Time spent in open arms of the EPM. Left: Measures obtained from SFR mice, the F2 generations of IMS fathers and mothers, the F2 generation of IMS mothers treated with theophylline (tph) during adolescent development, and the F3 and F4 generations of IMS mothers. Data are mean ± sem of 8 to 9 male animals/group. Significant differences revealed by AVOVA (F(5,53) = 3.058; p = 0.003)) were resolved *post hoc* using Tukey Kramer multiple comparisons tests. Right: F2 pups of IMS mothers were cross-fostered by SFR mothers (CF-F2) and IMS mothers raised SFR pups (RevCF-F2). Data are mean ± sem of measures from 7 male animals/group. Only RevCF-F2 mice spent less time in the open arms of the EPM (ANOVA, F(2,21) = 4.387, p = 0.027; *post hoc* Tukey-Kramer multiple comparisons tests)). (b) Immobility time in the FST. Left: Measures are obtained from SFR mice and the F2, F2-tph, F3, and F4 progenies of IMS mothers. ANOVA revealed significant differences between groups (F(5,53) = 8.9494; p = 0.0001)) that were resolved *post hoc* for F2 and F2-tph mice that exhibited significantly increased immobility. Right: Comparison of results obtained from cross-fostered mice. Significant differences revealed by ANOVA (F(2,22) = 7.181, p = 0.0045)) were resolved *post hoc* for RevCF-F2 mice that exhibited increase immobility. (c) EPM and FST behavior of the female F2 progenies of IMS mothers and fathers. Data (mean ± sem, N = 8 to 10 females/group) were compared by ANOVA (F(3,37) = 3.91; p = 0.01)) and significant differences were resolved *post hoc* using Tukey Kramer multiple comparisons tests as indicated.

We first examined whether the emotional phenotype resulting from early life stress is transmitted across generations. Adult male and female Balb/c mice that were exposed to the IMS paradigm were mated with non-stressed, standard facility reared (SRF) Balb/c mice to generate the F2 progenies that were then examined in adulthood. As shown in Fig. 1a and b, the F2 progeny of IMS mothers exhibited a similar emotional phenotype that characterizes the parental (F1) generation, namely increased anxiety-like behavior in the Elevated Plus Maze (EPM; Fig. 1a) and increased depression-like behavior in the Forced Swim Test (FST; Fig. 1b). In contrast, the emotional phenotype of the F2 progeny of IMS fathers did not significantly different from SFR controls (Fig. 1a, b). While the results shown in Fig. 1a and Fig. 1b were obtained from male F2 mice, Fig. 1c illustrates similar results obtained for the female F2 progenies, i.e., only offspring of IMS mothers exhibited decreased time in the open arms of the EPM and increased immobility in the FST. Hence, IMS Balb/c mothers transmit their emotional phenotype to both male and female offspring.

To test whether the maternal transmission of the IMS-triggered emotional phenotype is stable across generations, we assessed the behavior of F3 and F4 progenies of IMS mothers in the EPM and FST. While the F3 progeny still exhibited increased anxiety in the EPM (Fig. 1a), the behavior in the FST did no longer differ from SFR controls (Fig. 1b), and the EPM and FST behaviors of the F4 progeny were indistinguishable from SFR controls (Fig. 1a, b).

The fading emotional phenotype across generations of IMS mothers suggests that its transmission to the F2 progeny is germ-line independent. To test whether this transmission is mediated by maternal behavior during postnatal care of their pups, the F2 progeny of IMS mothers was cross-fostered to SFR mothers at the time of birth (CF-F2), and F2 progeny of first-time SFR mothers was raised by IMS mothers (RevCF-F2). Indeed, these F2 progenies adopted the behavioral phenotype of their cross-fostering mothers: The CF-F2 progeny exhibited normal behavior in the FST and EPM, but the RevCF-F2 progeny exhibited increased anxiety-like and depression-like behavior in the EPM and FST, respectively (Fig. 1a, b).

### The molecular phenotypes of the progenies of IMS mothers and fathers

We previously showed that IMS Balb/c mice have decreased activity of several class I/II HDACs, and that the quantitatively largest outcome of this is increased acetylation of histone H4K12 in the forebrain neocortex^8^. Moreover, we showed that adolescent treatment of IMS mice with an ultra-low dose theophylline (10^−5^ M), a dose that activates HDACs 1 and 3 but exerts no effects on adenosine receptors or phosphodiesterases^9^, effectively lowered H4K12 acetylation (without affecting H4K5 and acH4K8 acetylation)^8^. Lowering acH4K12 levels in IMS mice, however, worsened the IMS-triggered emotional phenotypes measured with the FST and EPM^8^. Strikingly, the same effect was detected in theophylline-treated F2 offspring of IMS Balb/c mothers: Compared with non-treated F2 mice, they exhibited significantly decreased time spent in the open arms of the EPM (Fig. 1a) and significantly increased immobility in the FST (Fig. 1b).

**Figure 2:**
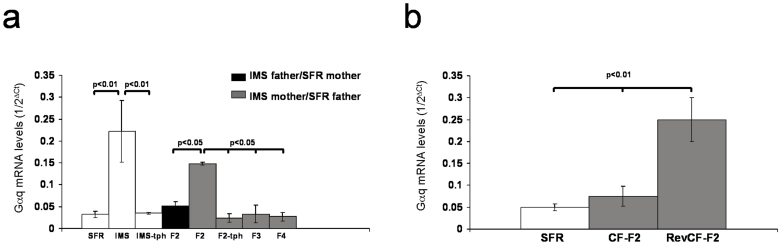
Maternal transmission of increased Gαq expression. (a) Data are mean ± sem (N = 6–8/group; equal numbers of males and females) of real-time RT-PCR measures. A comparison between SFR, IMS, and theophylline-treated IMS (IMS-tph) Balb/c mice (ANOVA (F(2,18) = 8.338; p = 0.0033; *post hoc* Tukey-Kramer multiple comparisons)) revealed significantly increased Gαq mRNA expression in IMS Balb/c mice that was reversed by adolescent theophylline treatment. Further comparison between SFR, F2 progenies of IMS fathers and mothers, F2 progenies of IMS mothers treated with theophylline (F2-tph), and F3 and F4 progenies of IMS mothers mice revealed significant differences (ANOVA (F(5,45) = 3.234; p = 0.0152) that were resolved *post hoc* only for the F2 progeny of IMS mothers that exhibited increased Gαq mRNA expression. (b) Gαq mRNA levels in cross-fostered mice. Significant differences revealed by ANOVA, F(2,17) = 12.22, p = 0.0007)) were resolved *post hoc* for F2-RevCF mice that exhibited increased Gαq expression.

These data suggest that the F2 progeny of IMS females might also exhibit increased acetylation of H4K12, an epigenetic mark of open chromatin and active gene transcription that is likely responsible for increased expression of distinct genes in IMS mice, including the increased Gαq expression described in previous studies^5^,^6^. To test this, we first used real-time PCR to compare forebrain neocortical Gαq mRNA expression levels between SFR, IMS, and F2 to F4 progenies of IMS mice. As shown in Fig. 2a, IMS Balb/c mice exhibit a ~4-fold increase in Gαq mRNA expression that was completely reversed by adolescent treatment with the HDAC-activating dose of theophylline. The F2 progeny of IMS mothers (but not fathers) also exhibited a theophylline-reversible increase in Gαq mRNA expression, but their F3 and F4 progenies reverted to normal Gαq mRNA expression (Fig. 2a). Moreover, similar to the emotional behavior of cross-fostered F2 mice, F2 mice raised by SFR mothers had normal Gαq mRNA expression levels, but Gαq mRNA levels of SFR pups raised by IMS mothers were significantly increased (Fig. 2b).

**Figure 3:**
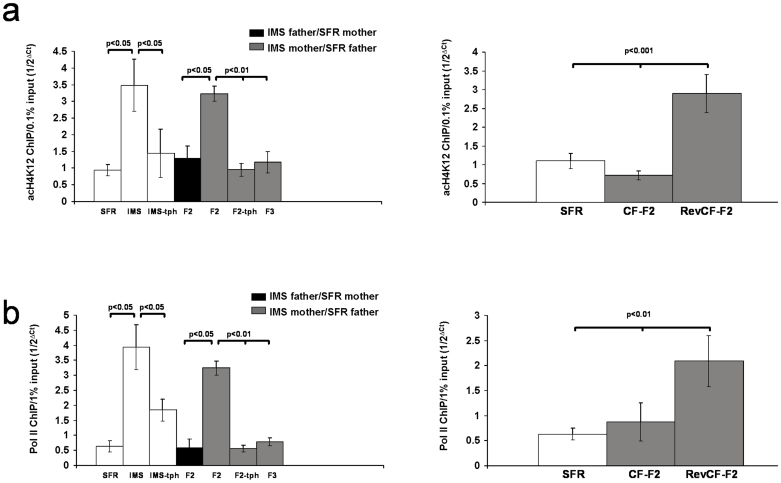
acH4K12 and Pol II ChIPs targeting the Gαq promotor. (a) acH4K12 ChIP. Left: A comparison between SFR, IMS, and IMS-tph mice (N = 9–10 animals/group; females and males) by ANOVA (F(2,27) = 4.958; p = 0.015; *post hoc* Tukey-Kramer multiple comparisons)) revealed significantly higher levels of acH4K12 in IMS Balb/c mice that were theophylline-reversible. A further comparison between SFR, F2 progenies of IMS fathers and mothers, and the F3 progeny of IMS mothers (N = 6–7 male and female animals/group) revealed significant differences (ANOVA (F(4,33) = 7.91; p = 0.0002) that were resolved *post hoc* only for the F2 progeny of IMS mothers. Their increased levels of acH4K12 were also theophylline-reversible. Right: Corresponding results obtained from cross-fostered mice. Significant differences revealed by ANOVA, F(2,18) = 14.459, p = 0.0003)) were resolved *post hoc* for RevCF-F2 mice that exhibited significant enrichment of acH4K12. (b) Pol II ChIP. Left: A comparison between SFR, IMS, and IMS-tph mice ((N = 6 animals/group; 3 females and 3 males) by ANOVA (F(2,18) = 11.455; p = 0.0007; *post hoc* Tukey-Kramer multiple comparisons)) revealed significantly higher levels of Pol II in IMS Balb/c mice that were theophylline-reversible. Another comparison between SFR mice, F2 progenies of IMS fathers and mothers, and the F3 progeny of IMS mothers (N = 5 males and females/group) revealed significant differences (ANOVA (F(4,25) = 29.52; p = 0.0001) that were also resolved *post hoc* only for the F2 progeny of IMS mothers that exhibited increased levels of Pol II at the Gαq gene promotor. This increased density of Pol II was not detected in the F2-tph progeny. Right: For the groups of cross-fostered mice and their SFR control, ANOVA (F(2,18) = 12.22, p = 0.0007)) revealed significant differences that were also resolved *post hoc* for RevCF-F2 mice that exhibited significantly greater Pol II enrichment.

The finding that increased expression of Gαq mRNA in IMS Balb/c mice and the F2 progeny of IMS females was reversed by adolescent treatment with an HDAC-activating dose of theophylline suggested that increased enrichment of acH4K12 at the promotor of the Gαq gene leads to increased gene transcription. To test this, chromatin immunoprecipitations (ChIPs) coupled with real-time PCR were performed using an anti-acH4K12 antibody in conjunction with primers targeting three non-overlapping genomic sequences of the Gαq gene that are located upstream of the transcription start site (see Methods and Supplementary Figure S1). From those three regions, the most distal region revealed an ~3-fold enrichment of acH4K12 in the forebrain neocortex of IMS Balb/c mice that was reversed by adolescent theophylline treatment (Fig. 3a). A similar enrichment was found in the F2 progeny of IMS mothers, and it was also theophylline-reversible (Fig. 3a). In contrast, neither the F2 progeny of IMS fathers nor the F3 progeny of IMS mothers exhibited increased enrichment of acH4K12 at this promotor sequence (Fig. 3a). Moreover, the levels of acH4K12 associated with the Gαq promotor in the F2 progenies of SFR and IMS mothers that were cross-fostered by IMS and SFR mothers, respectively, paralleled their Gαq mRNA expression levels, i.e., they were increased in RevCF-F2 mice, but indistinguishable from SFR controls in CF-F2 mice (Fig. 3a).

Hyperacetylation of H4K12 elevates the general accessibility of regulatory sequences to the transcriptional machinery, and the Gαq promotor sequence associated with increased acH4K12 enrichment contains a CCAAT box sequence that signals transcription factor-initiated recruitment RNA polymerase II (Pol II). Indeed, ChIP experiments revealed increased enrichment of Pol II in IMS mice and the F2 progeny of IMS mothers (Fig. 3b). In contrast, Pol II density was unaltered in the F2 progeny of IMS fathers and the F3 progeny of IMS mothers (Fig. 3b). It was also unaltered in F2 mice raised by SFR mothers, but increased in RevCF-F2 mice that were raised by IMS mothers (Fig. 3b).

To further test whether increased Pol II enrichment is acH4K12 dependent, we lowered acH4K12 levels in IMS mice and F2 mice of IMS mothers by treating them with theophylline as described above. Indeed, this treatment lowered Pol II enrichment at the Gαq promotor to levels indistinguishable from SFR controls (Fig. 3b).

Of note, although IMS Balb/c mice also exhibit increased acetylation of H4K5 and H4K8 protein^8^, there was no enrichment of either acetylated H4 variant at the promotor of the Gαq gene in the F2 progeny of IMS mothers (not shown), further supporting a specific relationship between increased acH4K12 levels and increased transcription of the Gαq gene. Altogether, these data support a functional link between enrichment of acH4K12 at the Gαq promotor and increased expression of Gαq mRNA in IMS Balb/c mice, and they demonstrate the propagation of this molecular phenotype to the F2 generation, but not to later generations, of IMS mothers.

### The cognitive phenotypes of theophylline-treated IMS mothers and their progenies

We previously showed that adult IMS Balb/c mice also have deficits in executive cognitive functions, specifically deficits in spatial working memory and extra-dimensional attention set-shifting, functions that are governed by the prefrontal cortex^7^. Since a role of Gαq-mediated signaling in working memory has previously been proposed^10^,^11^,^12^, we asked whether the cognitive deficits found in IMS mice are functionally linked to the epigenetic phenotype that increases Gαq expression and, if so, whether these deficits are also transmitted to the F2 progeny of IMS Balb/c mothers.

**Figure 4:**
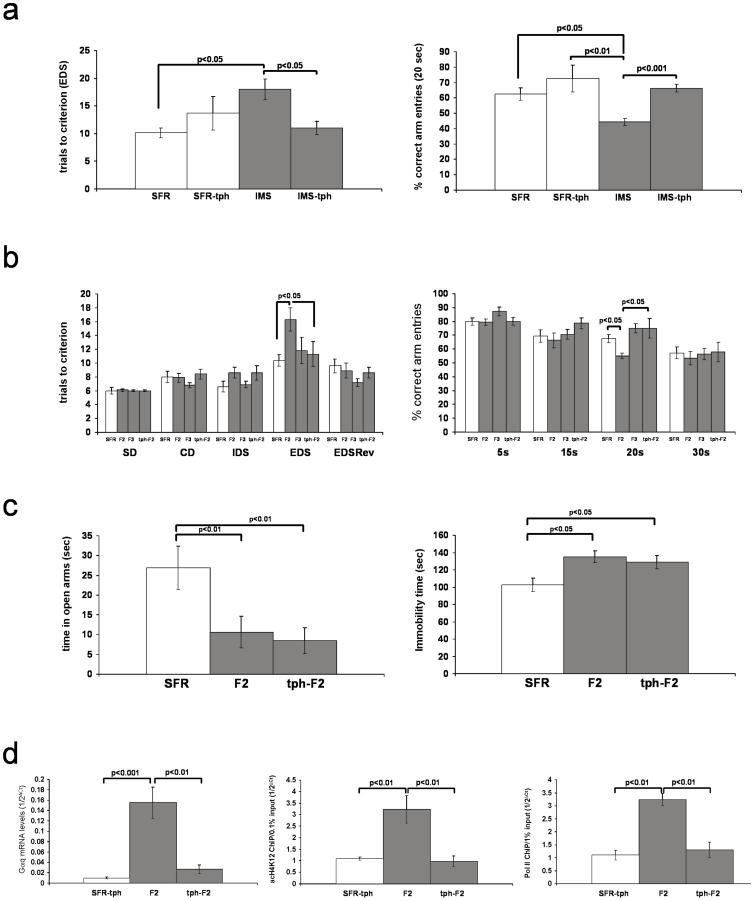
Effect of adolescent theophylline. (a) Effect on IMS mice. Left: ASST performance of non-treated and theophylline-treated SFR and IMS Balb/c mice. Right: WM performance. Date are mean ± sem of 6 to 8 mice/group. Statistical differences revealed by ANOVA were resolved *post hoc* (Tukey Kramer multiple comparisons) as indicated. (b) ASST and WM performance of SFR Balb/c mice, the F2 and F3 progenies of non-treated IMS mothers, and the F2 generation of theophylline-treated IMS Balb/c mothers (tph-F2). Data are mean ± sem of 8 to 12 animals per group. Statistical differences revealed by ANOVA (ASST: F(3,38) = 3.321, p = 0.03; WM: F(3,9) = 5.269, p = 0.006) were resolved *post hoc* (Tukey Kramer multiple comparisons) as indicated. (c) EPM (left) and FST behavior (right) of the F2 progeny of theophylline-treated IMS Balb/c mothers (tph-F2) compared to results obtained from SFR mice and the F2 progeny of non-treated IMS Balb/c mothers. Data (N = 11–12/group) were compared by ANOVA (EPM: F(2,35) = 5.374, p = 0.0095; FST: F(2,66) = 5.296, p = 0.0099) and statistical differences were resolved *post hoc* using Tukey Kramer multiple comparisons as indicated. (d) Gαq mRNA expression (left), acH4K12 (middle) and Pol II enrichment at the Gαq promotor (right) in the F2 progeny of theophylline-treated IMS Balb/c mice compared to theophylline-treated SFR mice and the corresponding results obtained from the F2 progeny of non-treated IMS Balb/c mothers shown in Fig. 2. For Gαq mRNA levels ANOVA revealed significant differences between the groups that were resolved *post hoc* (Tukey Kramer multiple comparisons) only for F2 mice of non-treated IMS mothers that exhibited significantly increased Gαq mRNA levels. Similarly, only F2 mice of non-treated IMS Balb/c mothers exhibited increased enrichment of acH4K12 and Pol II at the Gαq promotor. Data are mean ± sem of 7 (Gαq mRNA) and 5 to 6 animals per group (acH4K12 and Pol II ChIPs).

We first examined adult SFR and IMS Balb/c mice that were treated during adolescence with theophylline, and we compared their performance in tests of attention set-shifting (ASST) and spatial working memory (WM) with non-treated SFR and IMS mice. In the ASST, mice proceed through a series of test phases in which they discriminate between different odors and textures to find a food reward. Correct response selections are guided by one of two stimulus dimensions (texture and odors). In an intra-dimensional shift of attention (IDS), only the stimulus property of the guiding stimulus dimension is changed (i.e., one odor is switched for another odor), but in the extra-dimensional shift (EDS), the previously guiding stimulus dimension (i.e., odor) becomes irrelevant and the previously irrelevant dimension becomes relevant (i.e., texture). This is typically the most difficult test phase of the ASST, and we have previously shown that IMS Balb/c mice have deficits only in this test phase^7^. Indeed, as shown in Fig. 4a, the four groups of mice differed only in their performance the EDS phase (ANOVA, F(3,28) = 4.145, p = 0.016)): While non-treated IMS mice required more trials to criterion compared with SFR controls, the EDS performance of theophylline-treated IMS mice was significantly improved (and did not differ from SFR controls). This improvement cannot be explained by a “pro-cognitive” effect of theophylline itself since the EDS performance of non-treated and theophylline-treated SFR mice was not significantly different (Fig. 4a).

For IMS Balb/c mice, deficits in spatial WM were previously detected in a delayed alternation task (performed in a T maze) with 20 sec inter-trial delay^7^. A comparison of the WM performance of non-treated and theophylline-treated SFR and IMS mice also revealed significant differences only for the 20-sec delay period (ANOVA, F(3,29) = 10.23; p = 0.0001)) (Fig. 4a). Again, only non-treated IMS mice exhibited a significantly lower percentage of correct arm entries compared with SFR controls and theophylline-treated IMS mice, and the performance of non-treated and theophylline-treated SFR mice did not significantly differ.

Next we examined whether non-treated and theophylline-treated IMS Balb/c mothers transmit the IMS-triggered cognitive deficits to their progenies. As shown in Fig. 4b, the F2 progeny of non-treated IMS mothers also exhibit deficits in the EDS phase of the ASST and a WM deficit at 20 sec inter-trial delay. These deficits were no longer present in the F3 progeny of non-treated IMS mothers and, strikingly, these deficits were also not detected in the F2 progeny of theophylline-treated IMS mothers (labeled tph-F2) (Fig. 4b), although this progeny still had the same emotional phenotype that characterizes the F2 progeny of non-treated IMS mothers, namely increased anxiety-like behavior in the EPM and increased depression-like behavior in the FST (Fig. 4c).

If there is functional link between increased levels of acH4K12 (and associated gene expression changes) and deficits in WM and attention set-shifting, the tph-F2 progeny should also differ from the F2 progeny of non-treated IMS mothers in these molecular parameters. Hence, we compared the Gαq mRNA expression levels and acH4K12 and Pol II enrichments at the Gαq promotor between theophylline-treated SFR mice, the F2 progeny of non-treated IMS mothers and the thp-F2 progeny. For Gαq mRNA levels, ANOVA (F(2,21) = 13.661; p = 0.0002) revealed significant differences between these groups of mice that were resolved *post hoc* only for the F2 progeny of non-treated IMS mothers that exhibited increased Gαq mRNA levels. Hence, in the tph-F2 progeny, Gαq mRNA levels are no longer elevated above control levels (Fig. 4d). Similarly, there was a significant difference in acH4K12 enrichment at the Gαq promotor between theophylline-treated SFR mice (which did not significantly differ from non-treated SFR mice; p = 0.4) and the F2 progenies of non-treated and theophylline-treated IMS mothers (ANOVA, F(2,16) = 11.049, p = 0.0019), and *post hoc* multiple comparisons revealed that in tph-F2 mice, acH4K12 levels were significantly lower compared with F2 mice of non-treated mothers (Fig. 4d). The same held true for the enrichment of Pol II at the same promotor region (ANOVA, F(2,16) = 11.256; p = 0.0015) (Fig. 4d).

In summary, the F2 progeny of non-treated IMS Balb/c mothers exhibits the same executive cognitive deficits that were acquired by the parental generation that was exposed to early life stress. Lowering the levels of acH4K12 during adolescent development of IMS Balb/c mice blocks the emergence of executive cognitive deficits. Moreover, although theophylline-treated IMS mothers still transmit their emotional phenotype to the F2 progeny, this F2 progeny does also not exhibit executive cognitive deficits. Hence, removal of the cognitive deficits in the mother prevents the re-occurrence of such deficits in their F2 progeny.

### The phenotypes of fluoxetine-treated IMS Balb/c mothers and their F2 progeny

**Figure 5:**
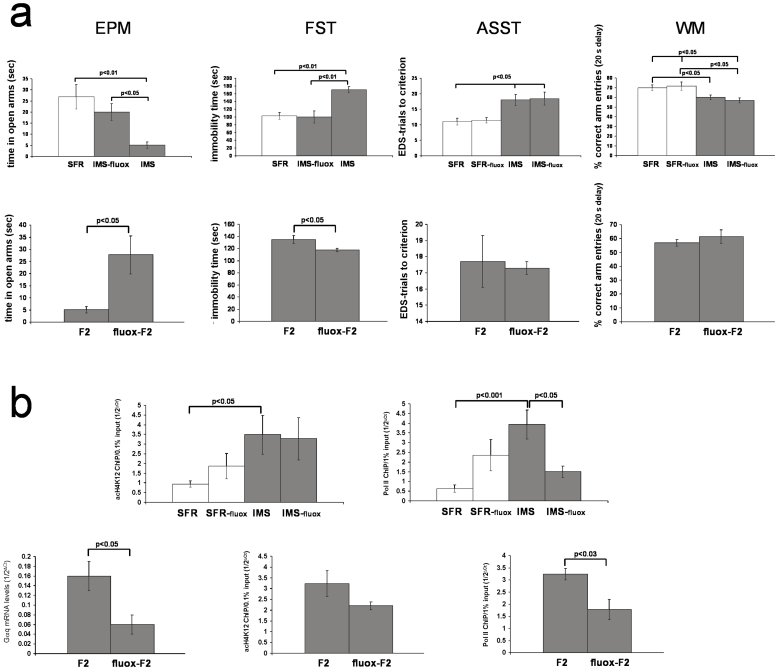
Effects of adolescent fluoxetine. (a) The effect of adolescent fluoxetine on emotional and cognitive behavior of IMS Balb/c mice and the F2 progeny of fluoxetine-treated IMS Balb/c mothers. Top: Effect on IMS Balb/c mice. Data are mean ± sem of 8 to 9 (EPM and FST) and 6 to 7 animals/group (ASST and WM). Statistical differences revealed by ANOVA were resolved *post hoc* using Tukey Kramer multiple comparisons tests as indicated. Bottom: Corresponding test results obtained from the F2 progeny of fluoxetine-treated IMS Balb/c mothers (labeled fluox-F2, N = 9 (EPM), N = 12 (FST), and N = 10 (ASST and WM) compared to results from the F2 progeny of non-treated IMS mothers (shown in Fig. 1 and 3) using two-tailed Students' *t* tests. (b) Effect of fluoxetine on acH4K12 and Pol II enrichment at the Gαq promotor of IMS Balb/c mice and the F2 progeny of fluoxetine-treated IMS Balb/c mothers. Top: Effect on SFR and IMS Balb/c mice. Data are mean ± sem of 7 to 8 (acH4K12 ChIP) and 5 to 6 animals per group (Pol II ChIP). ANOVA revealed significant differences between groups (acH4K12 ChIP: F(3, 29) = 3.73, p = 0.024; Pol II ChIP: F(3, 22) = 8.24, p = 0.001) that were resolved *post hoc* (Tukey Kramer multiple comparisons) as indicated. Bottom: Gαq mRNA levels (n = 8) and acH4K12 and Pol II enrichment at the Gαq promotor (N = 5) in fluox-F2 mice compared to corresponding results obtained from the F2 progeny of non-treated mothers that are shown in Fig. 2 (two-tailed Student's *t* tests).

We previously showed that, opposite to the effect of theophylline, adolescent fluoxetine treatment of IMS Balb/c mice further increased their levels of acetylated H4K12^8^. Consistent with our conclusion that increased acetylation of histone H4K12 is an epigenetic response to IMS that ameliorates the severity of the emotional psychopathology, fluoxetine-treated IMS Balb/c mice exhibited significantly improved emotional behaviors: In the EPM, fluoxetine-treated IMS mice spent significantly more time in the open arms compared with non-treated IMS mice, and their EPM behavior did no longer differ from SFR controls (ANOVA, F(2,26) = 7.863, p = 0.0024) (Fig. 5a). Similarly, in the FST, fluoxetine-treated IMS mice exhibited significantly less immobility compared with non-treated IMS mice, and their behavior was indistinguishable from SFR controls (ANOVA, F(2,24) = 8.746, p = 0.0016) (Fig. 5b).

A different result was obtained when the performance of non-treated and fluoxetine-treated SFR and IMS mice in the ASST and the WM test was compared. As shown in Fig. 5a, non-treated and fluoxetine-treated IMS mice showed the same deficits in the EDS phase of the ASST compared to both non-treated and fluoxetine-treated SFR mice (ANOVA, F(3,27) = 5.507; p = 0.0006) (Fig. 5a). Similarly, in the spatial WM test, fluoxetine-treated and non-treated IMS mice exhibited the same WM deficit at 20 sec delay compared with both non-treated and fluoxetine-treated SFR mice (ANOVA, F(3,270 = 4.183, p = 0.017) (Fig. 5a). Hence, fluoxetine-treated IMS Balb/c mothers differ from non-treated IMS mothers only in their emotional phenotype (which is indistinguishable from SFR controls).

Figure 5a (bottom) summarized the behavioral phenotypes of the F2 progeny of fluoxetine-treated IMS Balb/c mothers. Compared to the F2 progeny of non-treated IMS Balb/c mothers, the F2 progeny of fluoxetine-treated IMS mothers (labeled fluox-F2) spent significantly more time in the open arms of the EPM and displayed significantly less immobility in the FST. Yet, both F2 generations had the same deficit in the EDS phase of the ASST and spatial WM deficit at 20 sec delay (Fig. 5a). Hence, removal of the emotional deficits in the mother prevents the re-occurrence of such deficits in their F2 progeny, but the cognitive deficits exhibited by the mothers are equally evident in their F2 progeny.

At difference to the effect of adolescent theophylline treatment of IMS Balb/c mice that only reduces the levels of acH4K12 protein, adolescent fluoxetine has more widespread effects on histone protein expression and their post-translational modifications. In the forebrain neocortex, fluoxetine increased the levels of total histone H3 and H4 protein as well as the levels of acetylation of histone H4K12, H4K8, and H3K9. It also increased the levels of trimethylated H3 protein^8^. Moreover, we previously showed that adolescent fluoxetine treatment of IMS Balb/c mice significantly lowers their levels of Gαq mRNA and protein^5^ despite their elevated acetylation of histone H4K12^8^. Indeed, as shown in Fig. 5b (bottom), also the F2 progeny of fluoxetine-treated IMS Balb/c mothers expresses significantly less Gαq mRNA compared to the F2 progeny of non-treated mothers. ChIP experiments targeting the Gαq promotor of non-treated and fluoxetine-treated SFR and IMS Balb/c mice revealed that, despite similarly increased enrichment of acH4K12 in non-treated and fluoxetine-treated IMS mice, the density of Pol II was significantly lower in fluoxetine-treated IMS mice (Fig. 5b, top); a finding recapitulated in the fluox-F2 progeny (Fig. 5b, bottom). These findings indicate that increased enrichment of acH4K12 at the Gαq promotor leads to increased Gαq mRNA expression only when accompanied by increased densities of Pol II at the same promotor region.

### The phenotypes of C57Bl/6 mice cross-fostered by Balb/c mothers during IMS exposure

Current evidence suggests that the effects of IMS on pups is largely triggered by maternal behavior^13^. However, whether the responsiveness of the pups to the behavior of IMS Balb/c mothers is influenced by the genetic background of the pups has never been examined. Hence, we cross-fostered C56Bl/6 pups to parturient Balb/c mothers at the time of birth and subjected them to the IMS protocol. We chose the C57Bl/6 strain because we have previously shown that this strain is resilient to IMS in terms of their adult emotional and cognitive behavior^7^ and their epigenetic response (and related gene expression changes) when raised by C57Bl/6 mothers^6^,^12^.

First we tested whether the behavior of Balb/c mothers towards their own pups differs from their behavior towards their cross-fostered C57Bl/6 pups during the period of IMS exposure. We scored the maternal behavior towards pups when they were placed into a new home cage in which their 3 to 7-day old litters were randomly scattered around. In the mothers, this triggers a characteristic sequence of behavior: 1. Relocating of pups to a future nest position. 2. Brief licking and grooming of pups without nursing. 3. Nursing of pups. The time of onset of these behaviors were significantly shorter in SFR mothers compared to IMS mothers raising either their biological pups or cross-fostered C57Bl/6 pups, with no significant differences between the latter two groups (Supplementary Table 1).

**Figure 6:**
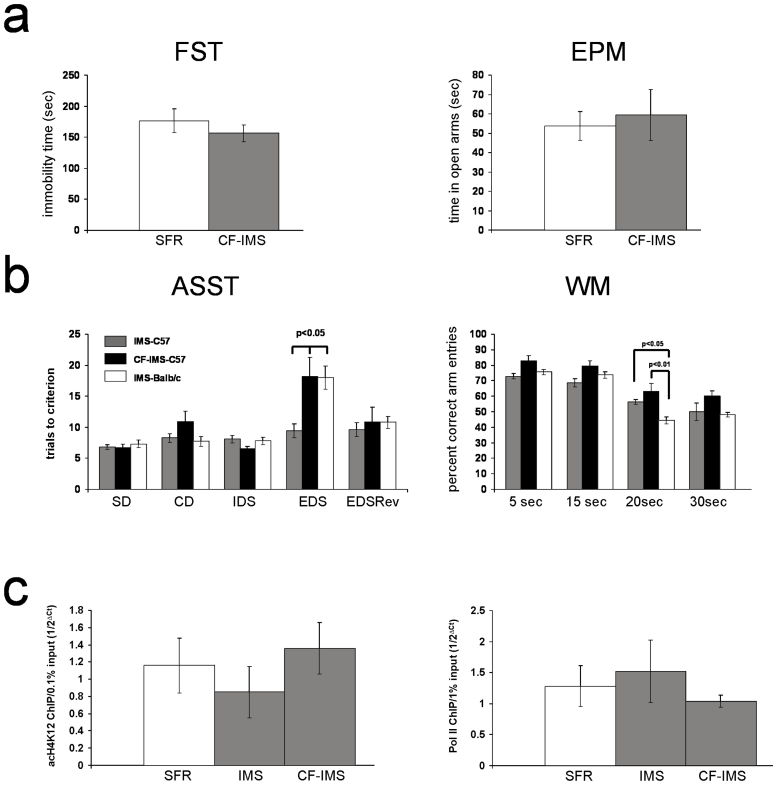
The phenotypes of cross-fostered IMS C57Bl/6 mice. (a) Behavior of cross-fostered IMS C57Bl/6 mice (CF-IMS) in the FST and EPM compared with SFR C57Bl/6 controls. Data are mean ± sem (N = 8/group, equal number of male and females). Two-tailed Students' *t* tests revealed no significant differences between the two groups. (b) ASST (EDS phase) and WM (20 sec delay) performance of cross-fostered IMS C57Bl/6 mice compared with IMS C57Bl/6 raised by C57Bl/6 mothers and IMS Balb/c mice raised by IMS Balb/c mothers. Each group is comprised of 7 animals that were tested in parallel. For the EDS phase of the ASST, significant differences revealed by ANOVA were resolved *post hoc* (Tukey Kramer multiple comparisons) for both CF-IMS-C57 and IMS-Balb/c mice that required significantly more trials to criterion compared with IMS C57Bl/6 mice. For the WM test at 20 sec delay, however, *post hoc* statistics revealed that only IMS Balb/c mice had a significantly lower percentage of correct arm entries compared with IMS C57 mice and CF-IMS-C57 mice. (c) acH4K12 and Pol II Chip targeting the Gαq promotor of SFR C57 mice, IMS C57 mice and CF-IMS-C57 mice. Data are mean ± sem of 5 to 6 animals per group (males and females). For either ChIP, ANOVA revealed no significant differences between these groups (acH4K12 ChIP: F(2,15)-0.8018, p = 0.5; Pol II ChIP: F(2, 18) = 0.1528, p = 0.9).

Despite unaltered behavior of Balb/c mothers to cross-fostered C57Bl/6 pups (labeled CF-IMS-C57Bl/6) during IMS exposure, these cross-fostered pups did not differ from SFR C57Bl/6 mice (raised by C57Bl/6 mothers) in their behavior in the FST and EPM in adulthood (Fig. 6a).

Next, we compared the ASST and WM performance of IMS-C57Bl/6 (raised by C57Bl/6 mothers), CF-IMS C57Bl/6 (raised by Balb/c mothers), and IMS Balb/c mice (raised by Balb/c mothers) (Fig. 6b). For the ASST, ANOVA revealed significant differences between these groups only for the EDS phase (F(2,21) = 6.619; p = 0.006)) in which CF-IMS C57Bl/6 mice, like IMS Balb/c mice, required significantly more trials to criterion compared with IMS C57Bl/6 mice. In the WM test, however, the performance of CF-IMS-C57Bl/6 mice did not significantly differ from IMS C57Bl/6 mice, and only IMS Balb/c mice differed significantly from both groups of C57Bl/6 mice in tests of 20 sec delay (ANOVA, F(2,21) = 8.158; p = 0.0028) in which they exhibited a significant deficit in spatial WM (Fig. 5B).

Finally, Fig. 6c summarizes the molecular phenotypes of CF IMS-C57Bl/6 mice. We previously showed that neither IMS C57Bl/6 mice nor CF-IMS-C57Bl/6 mice exhibit increased expression of Gαq mRNA in the adult forebrain neocortex^6^. Consistent with these earlier findings, neither IMS C57Bl/6 nor CF-IMS-C57Bl/6 mice exhibit increased acH4K12 or Pol II enrichment at the Gαq gene promotor.

In summary, prominent behavioral and molecular phenotypes of IMS exposure that develop in Balb/c mice and that are transmitted to the F2 progeny of IMS Balb/c mothers are largely absent in IMS C57Bl/6 mice that were raised by IMS mothers. One exception is a deficit in extra-dimensional attention set-shifting that is detected in both strains of IMS mice.

## Discussion

The present study shows that prominent behavioral and molecular phenotypes of Balb/c mice exposed to early life stress can be transmitted to the next generation. This includes anxiety- and depression-like behaviors in the EPM and FST, deficits in spatial working memory and extradimensional attention set-shifting, and increased enrichment of acH4K12 and RNA Polymerase II at the Gαq gene promotor leading to increased transcription of the Gαq gene. Although these phenotypes are found in males and females of the parental (F1) generation, transmission only occurs to the F2 progeny of IMS mothers, and affects male and female offspring equally.

A previous study showed that the effects of rodent neonatal separation result largely from alterations in the quality of maternal care and not from effects of separation *per se* on pups^13^. The present study indicates further that the transmission of IMS-evoked behavioral and molecular phenotypes to the F2 progeny is also triggered by maternal behavior: Offspring of IMS mothers that were adopted by SFR mothers at the time of birth do not exhibit the behavioral and molecular phenotypes of their biological mothers. Conversely, the emotional behavior and molecular measures of SFR pups raised by IMS mothers in the EPM and FST are similar to those of non-cross-fostered F2 progenies of IMS mothers. Yet, when the original stressor is removed, the phenotypes induced by the quality of maternal care do not last beyond a single generation (see also Ref. 14).

Trans-generational effects of maternal care phenotypes that lead to different DNA methylation phenotypes at a promotor region of the hippocampal glucocorticoid receptor gene in rats exposed to either high or low maternal care have first been reported by Weaver et al.^15^. In this study, a quantification of maternal care phenotypes allowed for the selection of extremes of high and low maternal care phenotypes for studies on the propagation of these phenotypes across generations. We have not attempted to select for extremes in the maternal behavior of our F2 mice towards the F3 progenies but the study of Weaver et al. suggests that, if an F2 Balb/c mother with an extreme low maternal care phenotype can be identified and used for further trans-generational studies, it is possible that trans-generational effects of early life stress can also be detected in the F3 progeny. Equally possible is that trans-generational effects of early life stress span more than just one generational cross when later progenies are also exposed to other forms of stress-precipitating life events, a possibility that remains to be investigated.

Earlier studies on the trans-generational effects of early life stress already found evidence for both germline-dependent and germline-independent propagation of early-life stress-triggered DNA methylation phenotypes^16^,^17^. The present study adds further evidence for a transmission of a specific histone-based epigenetic mark (hyperacetylation of histone H4K12) and tested how its erasure or augmentation in IMS mothers affected the phenotypes of these mothers and their F2 progeny. We found that an adolescent treatment that lowered the levels of acH4K12 in IMS Balb/c mothers augmented their emotional abnormality and that, conversely, an adolescent intervention that further augmented the levels of acH4K12 in IMS mice led to improved emotional behavior. These findings indicate that increased acetylation of histone H4K12 in the forebrain neocortex of IMS mice can ameliorate the severity of the adult emotional abnormalities. Strikingly and contrary to the effect on adult emotional behavior, lowering the acH4K12 levels in IMS Balb/c mice prevented the emergence of executive cognitive deficits. However, the adolescent intervention that further augmented the H4K12 acetylation left the executive cognitive deficits unaltered. Yet, since this treatment did not further impair these cognitive deficits, it remains still unresolved whether these deficits are truly acH4K12-dependent. Nevertheless, the finding that the F2 progenies of IMS mothers that underwent these adolescent interventions have the same behavioral and epigenetic phenotypes exhibited by their mothers indicates that each behavioral and epigenetic phenotype characteristic for early life stress exposure must be present in the mother to enable her to re-instate them in her offspring. In other words, treatments that prevent either the emergence of emotional or cognitive deficits in the mother also prevent the establishment of such deficits in her offspring. Moreover, the finding that treated mothers still transmitted their residual behavioral phenotype (emotional or cognitive) indicates that a germline-independent transmission of cognitive deficits is not dependent upon the presence of emotional deficits and *vice versa*. Hence, trans-generational effects of early life stress can only be abolished if both the emotional and the cognitive deficits of future mothers are effectively treated.

Acetylated H4K12 protein can exert gene-specific effects, and one example studied here is its role in stimulating the transcription of the Gαq gene. However, our data on fluoxetine-treated IMS Balb/c mice and the F2 progeny of fluoxetine-treated IMS Balb/c mothers indicate that, without concomitantly enhanced recruitment of RNA polymerase II to the promotor of the Gαq gene, enrichment of acH4K12 alone at the same promotor region is insufficient to stimulate gene transcription. Hence, despite the clear enrichment of acH4K12 at the Gαq promotor, the absence of a similar enrichment of Pol II suggests that fluoxetine treatment also triggers the establishment of other, yet to be identified epigenetic marks that can neutralize the effect of acH4K12. Indeed, we have previously shown that, at difference to adolescent theophylline, adolescent fluoxetine exerts more widespread effects on histone H3 and H4 protein expression and their post-translational modifications^8^.

Also, although the widespread expression of Gq protein in brain and the fact that about 40% of all G-protein-coupled receptors rely on signaling through Gq protein to stimulate phospholipid signaling^18^ tempted us to hypothesize that altered signaling through Gq-coupled receptors contributes to the IMS-triggered phenotypes, our data do not provide conclusive evidence that this is the case. At first glance, studies on non-treated and theophylline-treated IMS Balb/c mice and their F2 progenies could suggest that increased signaling through Gq-coupled receptors ameliorates the severity of the emotional abnormalities and/or triggers the emergence of cognitive deficits. However, our studies on fluoxetine-treated IMS Balb/c mice and the F2 progeny of fluoxetine-treated IMS Balb/c mothers show clearly that improved emotional behavior and the emergence of cognitive deficits can also occur in the absence of increased Gαq expression. Hence, the genes and signaling pathways affected by increased acetylation of H4K12 that are functionally linked to the emotional and cognitive phenotypes that emerge after early life stress exposure remain to be identified.

Finally, the finding that C57Bl/6 mice raised by Balb/c mothers from the time of birth and exposed to the IMS do not develop the epigenetic phenotype of early life stress exposure characteristic for IMS Balb/c mice illustrates that environmentally-triggered epigenetic responses are modulated by the genetic background. This is not surprising in view of recent evidence that links genetic (allelic) variations to variations in chromatin states^19^. Strikingly, however, although cross-fostered IMS C57Bl/6 mice exhibit normal emotional behavior and normal spatial working memory in adulthood, they still exhibit attention set-shifting deficits. This suggests that, contrary to the effect of the acH4K12 phenotype on emotional behavior and working memory, attention set-shifting deficits can emerge in the absence of this epigenetic phenotype. The molecular/epigenetic mechanisms leading to such set-shifting deficits in stress-resilient strains, however, remain to be discovered.

I summary, we begun to explore how adverse early life experiences of a parental generation influence subsequent generations, and we found new evidence for a germline-independent transmission of behavioral and epigenetic phenotypes of early life stress from mothers to their first progeny. Our finding that adolescent treatments that alter the epigenetic phenotype and, consequently, the behavioral phenotypes of future mothers lead to a progeny with similarly altered epigenetic and behavioral phenotypes implies that this maternal transmission -which confers risk for several mental disorders- can be prevented.

## Methods

### Animals

Balb/cJ and C57Bl/6J mice were housed in a temperature-controlled (26 ± 2°C) barrier facility with a 12-hour light/dark schedule (lights on at 8:00 am) and free access to food and water. All experiments were performed in accordance with the National Institutes of Health Guide for the Care and Use of Laboratory Animals and approved by the Institutional Animal Care and Use Committees at Columbia University and the New York State Psychiatric Institute.

### Infant maternal separation (IMS) and adolescent drug treatment

Offspring of first-time mothers were separated from their dam daily for three hours (from 1:00 to 4:00 pm) from postnatal age day 2 (P2) until P15. Control animals were standard-facility-reared (SFR) pups of first-time mothers. Housing and husbandry conditions were identical for IMS and SFR mice. Pups were weaned at postnatal age P28 and group-housed by sex (four to five animals randomly selected from 4 to 5 different litters). In this study, we refer to mice exposed to the IMS paradigm as the F1 generation. This F1 generation of adult male and female IMS mice was then mated with SFR mice to generate the F2 progeny, and adult F2 mice were used to generate the F3 and F4 generation of IMS mothers. All males were removed after mating.

Some of the F1 mice and the F2 progeny of IMS mothers were treated either with an ultra-low dose of theophylline that uniquely activates HDACs^9^ or with fluoxetine. These treatments were initiated at P35 (the age at which decreased HDAC expression and increased acetylation of histone H4 protein are first detected^8^ and terminated at P59. Both drugs were administered via the drinking water, and mice consumed ~10^−5^ M of theophylline or 12 to 16 mg/kg fluoxetine per 24 hours. At the end of these drug treatments, mice were either tested as described below, or female mice were mated with SFR males to generate the maternal F2 progeny of these drug-treated mice.

### Behavioral tests

#### Elevated Plus Maze (EPM)

Male and female mice were exposed to the Elevated Plus Maze (EPM) for 5 min. Their times spent in open arms and the numbers of open and closed arm entries were recorded.

#### Forced Swim Test (FST)

One week after exposure to the EPM, mice were tested in a modified version of the FST, i.e., a 6 min exposure on day 1 followed by another 6 min exposure on day 2. On both days, the number of passive episodes and their duration (in sec) were recorded during the last 4 min of FST exposure, and the results obtained from day 2 were compared between the different groups.

#### Attention-Set-Shifting Task (ASST)

Prior to testing in the ASST, mice (males and females) were food restricted such that they gradually (over the period of 5 to 7 days) lost 10% of their free-feeding body weights. First, mice learned to dig for food buried deeply in unscented terra cotta pots filled with familiar bedding medium. Then, they proceeded through 5 consecutive test phases of the ASST. The first phase is a simple discrimination (SD) between odor (scented terra cotta pots) or texture (different digging media) followed by a compound discrimination (CD) in which another stimulus property (a second odor or texture) was introduced that was not a reliable predictor of food reward. The next phase required an intradimensional shift of attention (IDS), i.e., both relevant and irrelevant stimulus properties were changed, but the relevant stimulus dimension used in the SD and CD (odor or medium texture) remained the same. Then, the formerly irrelevant stimulus dimension became relevant and required an extradimensional shift of attention (EDS). Finally, the rules of the EDS test phase were reversed (EDS-Rev). In all test phases, animals had to reach a criterion of 6 consecutive correct trials, and the number of trials to criterion was referred to as response accuracy.

#### Spatial Working Memory (WM)

Spatial WM was assessed with a delayed alternation task performed in a T-maze. Mice were trained for alternate arm entries in the T-maze until they reached 70% correct arm entries (in 10 trials per day) on 2 consecutive days with 5 sec inter-trial delay periods. Then, mice performed the test with 3 longer inter-trial delays (15, 20, and 30 sec, each tested on 2 consecutive days). Only correct arm entries were rewarded with food, and the percentage of correct arm entries in the total number of 10 trials per delay period was taken as a measure of response accuracy.

### RNA extraction and real-time PCR

Total RNA was extracted from dissected forebrain neocortical tissue using guanidine/cesium chloride ultracentrifugation. First-strand cDNA was synthesized using Murine Moloney Leukemia Virus reverse transcriptase (USB, Cleveland, OH) in conjunction with oligo dT_15_ primers. Real time PCR was performed using the iQ Real Time PCR detection System (Bio-Rad, Hercules, CA) and SYBR Green (Bio-Rad). Gαq cDNA was amplified using the primers 5′-ACTCTGGAGTCCATCATG3′/TGTATGGGATCTTGAGCG-3′. Cycle thresholds (Ct) of amplification (normalized to β-actin whose Ct values did not differ between groups) were expressed as 1/2^ΔCt^ values so that higher numbers reflect higher expression.

### Chromatin Immunoprecipitations (ChIP)

Forebrain neocortical tissue of male and female mice was fixed with 1% paraformaldehyde, dounce homogenized and sonicated using the Microson ultrasonic cell disrupter (Misonix, Farmingdale, NY)) to an average DNA length of 200 to 400 bp. Samples were centrifuged at 15,000 × g, and 50 μl aliquots of the supernatant were immunoprecipitated overnight with 20 μl protein A magnetic beads (Millipore, Temecula, CA) and 4 μg of ChIP-grade anti-acetyl histone H4 (Lys 12) antibody (Millipore) or 3 μg of a ChIP-grade antibody directed against RNA Polymerase II CTD repeat YSPTSPS (Abcam, Inc., Cambridge, MA). Beads were treated according to the instruction of the manufacturer. Immunoprecipitated DNA and a serial dilution of 1% input were analyzed by SYBR-Green real-time PCR.

Oligonucleotides used in the PCR reaction targeted a stretch of 621 nucleotides (numbered −1 to −621) of the Gαq gene located upstream of the most 5′ nucleotide of the RNA sequence (NCBI Reference sequence NM_008139.5). Primers targeted either the most proximal sequence (−1 to −240), the middle portion of the sequence (−281 to −474), or the distal sequence (−489 to −618). The following primer pairs amplified the targeted sequence in both input and ChIP DNA: 5′-GAGAGGCCCGGAGCGCA-3′/5′-CCTCCCTCTGTGCGAGCT-3′ (−281–474; annealing temperature: 62°C) and 5′-AGAGAGCCAGTGACAATC-3′/5′-GGGCTGCCCTTTCCTATT-3′ (-489-618; annealing temperature: 50°C). Cycle thresholds (Ct) of PCR amplification (45 cycles) were normalized to the Ct values obtained for 0.1% (acH4K12) or 1% input DNA (Pol II) and expressed as 1/2^ΔCt^.

## Author Contributions

C.S. designed the study, performed ChIP and PCR experiments, wrote the manuscript with all authors edited. Z.L.-Mc. and L.R.M. performed the behavioral experiments and analyzed these results.

## Supplementary Material

Supplementary Information Figure S1 and Table S1
