# Supplementary material for: Trans-generational Effects of Early Life Stress: The Role of Maternal Behavior
Source: Sci Rep. 2014 May 2;4:4873. doi: 10.1038/srep04873 (PMC4007084; doi:10.1038/srep04873)
Supplement: Supplementary Information — Figure S1 and Table S1 [file srep04873-s1.doc]

**SUPPLEMENTARY INFORMATION**

**SREP-14-00439**

**Trans-generational Effects of Early Life Stress: The Role of Maternal Behavior**

**Claudia Schmauss^1,2*^, Zoe Lee-McDermott^1^, and Liorimar Ramos Medina^1^**

*^1^Department of Psychiatry and Molecular Therapeutics, Columbia University and ^2^New York State Psychiatric Institute, New York, NY 10032*

**Content:**

1. Supplementary Figure 1
2. Supplementary Table 1


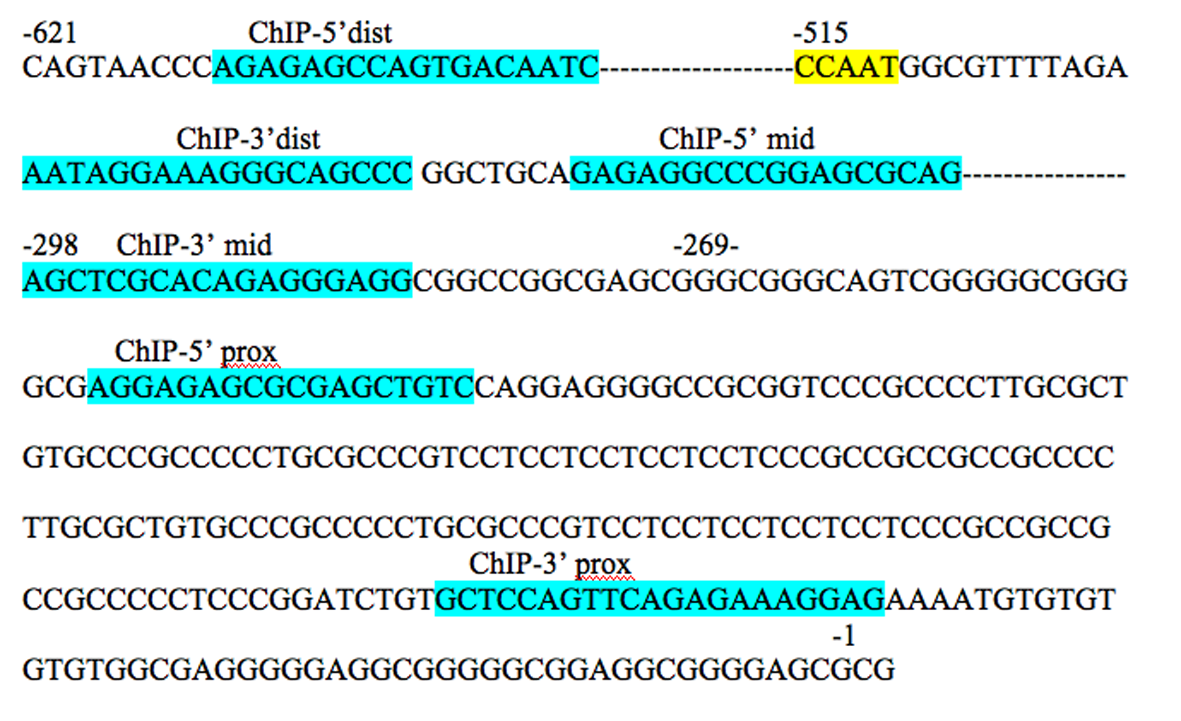


**Figure S1.** ***The promotor sequence of the Gαq gene.*** A CCAAT is box highlighted in yellow. ChIP primer sequences targeting the distal (dist), middle (mid) or proximal (prox) sequences (relative to the most 5’ nucleotide of the mRNA sequence) are highlighted.

**Table S1**. **Maternal behavior towards pups in a new home cage^*^**.

| **Scored Behavior** | **SFR** | **IMS (raising Balb/c)** | **IMS (raising C57Bl/6)** |
| --- | --- | --- | --- |
| *Pup relocation* | 167.3 ± 48.7 sec | 656.5 ± 84.0 sec^a^ | 590 ± 79.4 sec^a^ |
| *Licking/grooming (without nursing)* | 213.0 ± 57.5 sec | 1062.5 ± 115.4 sec^b^ | 1009 ± 91.3 sec^b^ |
| *nursing* | 722.5 ± 121.5 sec | 1605.0 ± 256.2 sec^c^ | 1400 ± 204.1 sec |

^*^Data are mean ± sem obtained from 4 litters per groups and were compared by ANOVA followed post hoc by Tukey-Kramer multiple comparisons tests. ^a^p<0.01, ^b^p<0.001, and ^c^p<0.05 compared with SFR.
